# Benzoic Acid in Cystitis

**Published:** 1877-07-20

**Authors:** 


BENZOIC ACID IN CYSTITIS.—In the Wayne county Medical Society, Michigan, Dr. Mulhorn reported a case of obstinate chronic cystitis relieved by ten grain doses of benzoic acid. He regards it an admirable remedy in cystitis.

